# Improved spectral resolution of [^13^C,^1^H]-HSQC spectra of aromatic amino acid residues in proteins produced by cell-free synthesis from inexpensive ^13^C-labelled precursors

**DOI:** 10.1007/s10858-023-00420-9

**Published:** 2023-06-20

**Authors:** Damian Van Raad, Thomas Huber, Gottfried Otting

**Affiliations:** 1 Research School of Chemistry, Australian National University, Canberra, ACT 2601 Australia; 2 ARC Centre of Excellence for Innovations in Peptide & Protein Science, Research School of Chemistry, Australian National University, Canberra, ACT 2601 Australia

**Keywords:** Amino acid metabolism, Aromatic amino acids, Cell-free protein synthesis, eCell, ^13^C-labelling

## Abstract

Cell-free protein synthesis using eCells allows production of amino acids from inexpensive ^13^C-labelled precursors. We show that the metabolic pathway converting pyruvate, glucose and erythrose into aromatic amino acids is maintained in eCells. Judicious choice of ^13^C-labelled starting material leads to proteins, where the sidechains of aromatic amino acids display [^13^C,^1^H]-HSQC cross-peaks free of one-bond ^13^C–^13^C couplings. Selective ^13^C-labelling of tyrosine and phenylalanine residues is achieved simply by using different compositions of the reaction buffers.

**Supplementary Information:**

The online version contains supplementary material available at 10.1007/s10858-023-00420-9.

## Introduction

The NMR spectroscopic analysis of proteins of increasing molecular weight is hindered by cross-peak overlap. Selective isotope-labelling presents an elegant way for improving the spectral resolution (Verardi et al. 2012) with site-selective ^13^C and ^2^H labelling delivering greatly simplified protein NMR spectra, which benefit the analysis of large proteins (Takeuchi et al. 2007; Kainosho and Güntert 2009; Takeda et al. 2010). As a drawback, the cost of sample preparation with isotope-labelled amino acids can be high (Gell et al. 2017). The present work presents a new strategy for selective ^13^C-labelling of aromatic amino acids that uses inexpensive ^13^C-labelled precursors to direct ^13^C labels into specific positions of the aromatic side chains of phenylalanine (Phe), tryptophan (Trp) and tyrosine (Tyr) in a cell-free protein synthesis (CFPS) system. NMR spectroscopy offers powerful means to assess the dynamics of aromatic amino acid side chains, and site-selective ^13^C-labels present particularly convenient probes (Akke and Weininger 2023).

Due to large one-bond ^13^C–^13^C couplings (^1^*J*_CC_) and limited chemical shift dispersion between the δ and ε CH groups of Tyr and δ, ε and ζ CH groups of Phe (Williams et al. 2015), the [^13^C,^1^H]-HSQC spectra of the aromatic rings of Phe, Trp and Tyr residues are prone to spectral overlap, when the proteins are produced with uniform ^13^C-labelling (Torizawa et al. 2005; Milbradt et al. 2015). Furthermore, ^1^*J*_CC_ couplings of aromatic side chains are large and hinder relaxation measurements to probe for conformational changes (Teilum et al. 2006; Kasinath et al. 2013). ^1^*J*_CC_ couplings also limit the gain in sensitivity and resolution that can be obtained from aromatic TROSY experiments (Pervushin et al. 1998; Milbradt et al. 2015). This situation has triggered the development of various atom-specific labelling methods to reduce the complexity of the ^13^C-NMR spectra of aromatic amino acids by eliminating ^1^*J*_CC_ couplings (Schörghuber et al. 2018). As the chemical synthesis of suitably ^13^C-labelled amino acids is expensive, a more cost-efficient approach is to harness the anabolic pathways of in vivo amino acid synthesis to produce specifically labelled protein from relatively inexpensive labelled precursors. In the case of Phe and Tyr, *E. coli* cell cultures in minimal media supplied with ^13^C-labelled α-ketoacids as late-stage precursors were shown to produce selectively labelled protein in vivo without cross-labelling (Lichtenecker et al. 2013; Lichtenecker 2014). Selectively ^13^C-labelled pyruvate and erythrose, which are earlier precursors of aromatic amino acids, have also been used successfully to label single δ-carbon sites in Phe and Tyr and the ε_3_-carbon in Trp (Kasinath et al. 2013).

In vivo labelling strategies are prone to isotope scrambling, which can be suppressed by limiting the time of bacterial cell growth after induction to prevent precursor recycling (Kurauskas et al. 2017). In an extension of this approach, selected amino acids can be “unlabelled” by their provision in the growth medium in their standard form, i.e., at natural isotopic abundance (Rasia et al. 2012; Lacabanne et al. 2018).

To obtain the protein quantities required for NMR spectroscopy, in vivo protein expression requires significant amounts of the isotope-labelled amino acids or precursors, whereas contemporary cell-free protein synthesis systems incorporate amino acids in much higher yield (Torizawa et al. 2004). Unfortunately, many of the bacterial enzymes are rendered nonfunctional during the preparation of cell extracts, so that the less expensive isotope labelling approaches that depend on multi-step anabolic pathways are inaccessible to conventional CFPS (Linser et al. 2014). As replenishing the cell-free extract with the requisite enzymes would be costly and unpractical, we explored the use of the recently developed eCell system for protein production under CFPS conditions. eCells are bacterial cells coated with layers of differently charged polyionic polymers and cell walls made porous by the expression of endolysin (Van Raad and Huber 2021). The pores of eCells leak proteins only very slowly over a period of hours, whereas low-molecular weight compounds readily exchange between the intra- and extracellular space. eCells can therefore be compared with traditional CFPS systems that use dialysis membranes separating inner and outer buffer solutions (Apponyi et al. 2008). Importantly, the mild preparation conditions of eCells maintain the activity of bacterial anabolic enzymes much better than cell extracts. In previous work we observed that, besides maintaining the entire repertoire of transcription/translation components, eCells also preserve the enzymes required for the biosynthesis of valine and leucine from pyruvate and glucose (Van Raad et al. 2023). The present work shows that eCells likewise preserve the fully functional complement of biosynthetic *E. coli* enzymes required for the synthesis of Phe, Trp and Tyr and therefore allow the production of proteins with site-specifically ^13^C-labelled aromatic amino acids from inexpensive precursors.

**Fig. 1:**
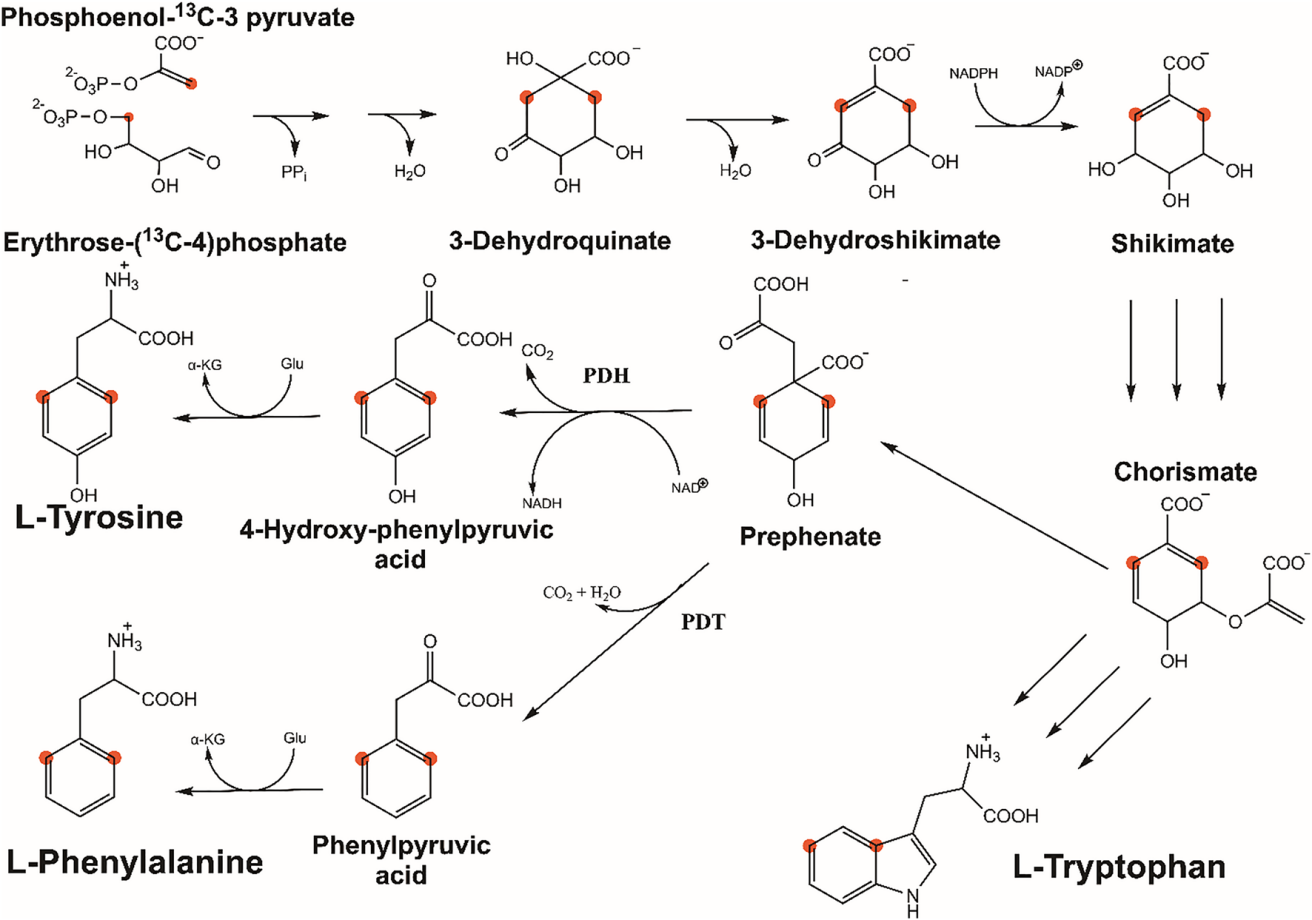
Biosynthetic pathway of the aromatic amino acids tyrosine, phenylalanine and tryptophan starting from phosphoenol-pyruvate and erythrose-4-phosphate. The present work used 4-^13^C erythrose and 3-^13^C pyruvate in eCells to produce proteins with specifically ^13^C-labelled aromatic amino acids. Red balls mark the ^13^C-enriched positions and trace their fate through the pathways

The bacterial biosynthetic pathways of aromatic amino acid synthesis (Fig. 1) show that pyruvate and erythrose are precursors that lead to the final amino acid product via a limited number of intermediates. The combination of phosphoenol pyruvate with erythrose phosphate produces Phe, Trp and Tyr, indicating that selective ^13^C-labelling of the δ and ε positions of Tyr/Phe and the δ_2_ position of Trp can be achieved in eCells by the use of ^13^C-labelled pyruvate and erythrose, in analogy to the situation in vivo (Lundström et al. 2007). The present work shows that this is indeed the case.

## Materials and methods

### Plasmids

The expression vectors used for *E. coli* peptidyl–prolyl *cis–trans* isomerase B (PpiB) and ubiquitin were described previously (Van Raad and Huber 2021). They afford expression of the target proteins under control of a T7 promoter and *lac* operator and contain a spectinomycin resistance gene.

### eCells

The production of eCells followed a previously published protocol, using *E. coli* autolysis Xjb(DE3)^*^ cells, which have an endolysin gene in the genome under a P_BAD_ promoter (Van Raad et al. 2021). The culture was induced for endolysin production at the time of inoculation with a final concentration of 3 mM arabinose. The cells were grown to OD_600_ 0.6 and washed three times with PBS-E (phosphate-buffered saline containing 137 mM NaCl, 2.7 mM KCl, 10 mM Na_2_HPO_4_, 1.8 mM KH_2_PO_4_ and 1 mM EDTA) pH 7.4 and resuspended in 0.25 mg/mL of chitosan in PBS-E solution. After vigorous shaking for 20 min, the cell pellet was washed with PBS-E pH 6.0 three times to remove excess chitosan and then resuspended in 0.25 mg/mL of alginate PBS-E solution and subjected to vigorous shaking for 20 min. The cells were then washed 3 times with PBS-E pH 6.0, resuspended in PBS-E pH 7.4 and directly stored at −80 ^o^C.

### CFPS

The protocol for eCell CFPS based on glucose and pyruvate has been described previously (Van Raad et al. 2023). The protocol was adapted from the phosphate recycling system published by Jewett and Swartz (2004). The CFPS buffer contained 0.9 mM UTP and CTP, 50 mM HEPES, 1.5 mM GTP, 1.5 mM ATP, 0.68 µM folinic acid, 0.64 mM cAMP, 1.7 mM DTT, 60 mM KGlu, 8 mM MgGlu, 2% v/v PEG-8000, 5 mM CoA, 33 mM pyruvate, 4 mM sodium oxalate, 0.25 mM CoA and 0.33 mM NAD. 3.5 mM of each amino acid was added except those targeted for isotope enrichment by the eCells. Na_2_HPO_4_ was omitted to promote the use of glucose for energy generation, and 4 mM sodium oxalate was used to promote the generation of ATP by the PANOx system of Jewett and Swartz (2004).

Prior to each CFPS reaction, the buffer was adjusted to pH 7.5. Frozen aliquots of encapsulated cells were thawed and the pellet immersed in CFPS buffer. Except where stated otherwise, 1.5 g eCells produced from 1 L cell culture at OD_600_ 0.6 were suspended in 25 mL CFPS buffer. CFPS was conducted for 12 h at 37 ^o^C in a shaker at 180 rpm. Proteins were extracted from the eCells by sonication for 2 min at 60 kHz and spinning down the resulting mixture for 15 min at 24,000 *g* prior to purification using His Gravitrap columns (GE Healthcare).

### Labelling with 3-^13^C pyruvate, 2-^13^C glucose or with 1-^13^C glucose and 4-^13^C erythrose combined

To produce ubiquitin samples from 3-^13^C pyruvate for ^13^C-labelling of the δ positions of tyrosine and phenylalanine, dry 3-^13^C pyruvate was added to 300 mg eCells in 10 mL CFPS buffer at 33 mM final concentration, and Tyr or Phe were omitted from the CFPS buffer.

To test the performance of 2-^13^C-glucose as carbon source in the aromatic amino acid synthesis, dry 2-^13^C glucose was added at 30 mM final concentration to 1.5 g eCells suspended in 25 mL CFPS buffer. Tyrosine, phenylalanine and tryptophan were omitted from the amino acid mixture to allow for labelling of the ε positions of tyrosine and phenylalanine and the tryptophan δ_2_ position.

For increased levels of ^13^C labelling of PpiB, 3-^13^C pyruvate was added to CFPS buffers at 33 mM final concentration combined with 4-^13^C erythrose at 30 mM concentration. Tyrosine and phenylalanine were omitted from the CFPS reaction mixture.

For increased ^13^C labelling of PpiB using pyruvate and an isotopologue of glucose, 3-^13^C pyruvate was added to CFPS buffer at 16.5 mM final concentration combined with 1-^13^C glucose at 16 mM concentration. Tyrosine and phenylalanine were omitted from the reaction mixture. For all samples, 1 mM IPTG was added to the CFPS reaction mixture.

### NMR spectroscopy and isotope labelling yields

[^13^C,^1^H]-HSQC spectra were recorded at 25 ^o^C using Bruker 600 or 800 MHz NMR spectrometers equipped with TCI cryoprobes. To determine the degree of isotope enrichment, the [^13^C,^1^H]-HSQC cross-peak integrals of the labelled residues were compared with those of an internal standard of 0.1 mM 3-^13^C pyruvate in spectra recorded with a recovery delay of 30 s between scans.

## Results

The biosynthesis of aromatic amino acids under CFPS conditions was explored using eCells made from *E. coli* XjB(DE3)* cells. The eCells contained the expression plasmids for ubiquitin or the *E. coli* prolyl *cis–trans* peptidyl isomerase B (PpiB). eCell CFPS supplied with 3-^13^C pyruvate is expected to label a single δ carbon of phenylalanine and tyrosine (Fig. 1), while for tryptophan the ^13^C label is expected to appear in the position of a quaternary carbon which does not produce a [^13^C,^1^H]-HSQC cross-peak.

**Fig. 2:**
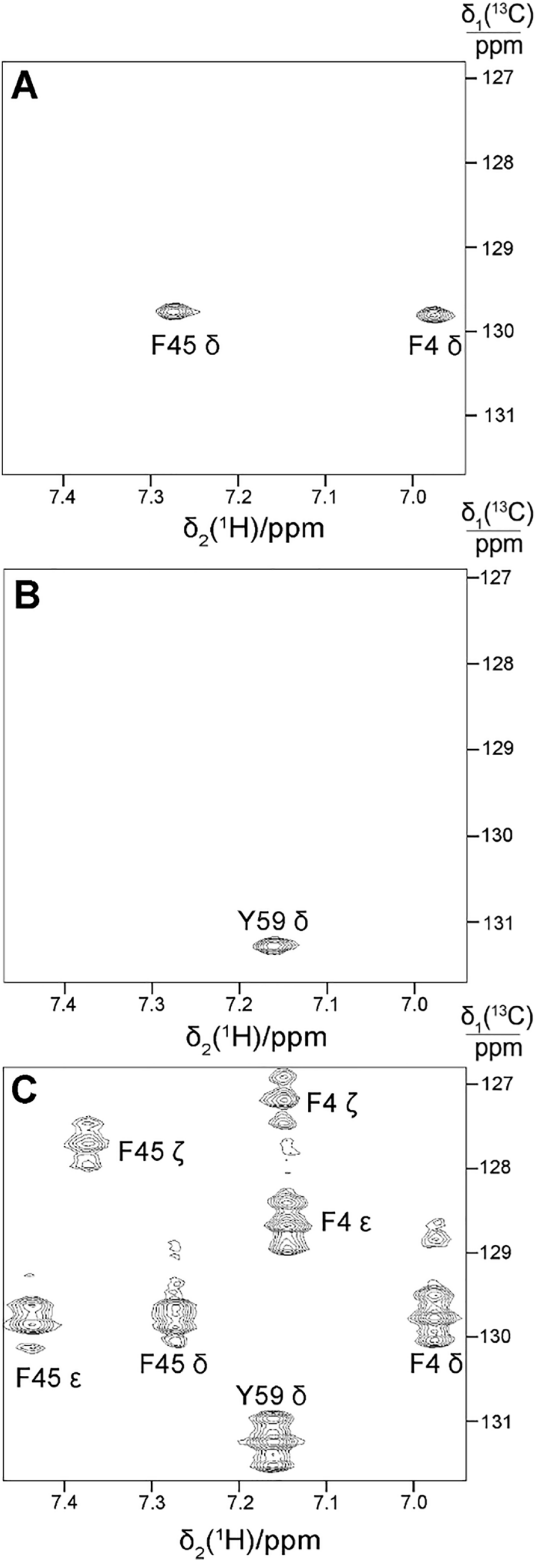
Selective ^13^C-labelling of the δ positions of phenylalanine or tyrosine in ubiquitin produced by eCell CFPS with 3-^13^C pyruvate as the carbon source. The plots show selected spectral regions from [^13^C,^1^H]-HSQC spectra using different labelling strategies. **A** C^δ^H cross-peaks of the two phenylalanine residues of ubiquitin. The protein was produced in the presence of 3.5 mM unlabelled amino acids, including Tyr but not Phe. Protein yield was 1.2 mg from 300 mg eCells in 10 mL CFPS buffer. **B** C^δ^H cross-peak of the single tyrosine residue of ubiquitin. The protein was produced in the presence of 3.5 mM unlabelled amino acids, including Phe but not Tyr. Protein yield 0.8 mg. **C** Uniformly ^13^C-labelled sample prepared using uniformly ^13^C-labelled glucose in a 5 mL eCell CFPS reaction containing 300 mg eCells, omitting Tyr and Phe. The yield was 1.7 mg purified protein

The NMR spectra of ubiquitin confirmed these expectations. Ubiquitin contains two Phe, one Tyr and no Trp residue. As expected, two [^13^C,^1^H]-cross-peaks are observed for the sample prepared with Phe omitted and unlabelled Tyr added in the CFPS buffer (Fig. 2A), whereas a single cross-peak is observed for the sample prepared with Tyr omitted and Phe added (Fig. 2B). As anticipated, these cross-peaks show no evidence of ^1^*J*_CC_ splittings, which are prominent in uniformly ^13^C-labelled samples (Fig. 2C). 300 mg eCells suspended in 10 mL buffer containing 33 mM 3-^13^C pyruvate were sufficient to produce 1.2 mg Phe ^13^C^δ^-labelled or 0.8 mg Tyr ^13^C^δ^-labelled purified ubiquitin with isotope labelling levels of 37% and 40%, respectively. In principle, using 3-^13^C pyruvate allows for a labelling degree of up to 50%, and we attribute the somewhat reduced isotope enrichment to a residual reservoir of unlabelled amino acids present in the eCells. We attempted to increase the level of isotope labelling by extensive dialysis of the eCells in phosphate buffered saline prior to use in CFPS, but this did not significantly increase the level of labelling. Most importantly, the presence of unlabelled Tyr or Phe did not affect the selectivity of labelling.

To demonstrate the general applicability of the labelling protocol, a sample of PpiB was made using 33 mM 3-^13^C pyruvate and 1.5 g eCells. 4.8 mg PpiB was obtained with a labelling level of about 36%. To increase the labelling degree of the C^δ^ positions, we also prepared a sample using 16 mM 1-^13^C glucose and 16.5 mM 3-^13^C pyruvate. The experiment yielded 2.7 mg protein per gram of eCells with about 50% ^13^C labelling of the C^δ^ positions. The [^13^C,^1^H]-spectra of the C^δ^-labelled preparations showed resolved cross-peaks for 10 Phe and 3 Tyr residues free of multiplet splittings due to ^1^*J*_CC_ couplings (Figures S1 and S2). PpiB contains 12 Phe and 3 Tyr residues, but some of them undergo ring flips in the intermediate time regime, where signals are broadened beyond detection (Takeda et al. 2010).

**Fig. 3:**
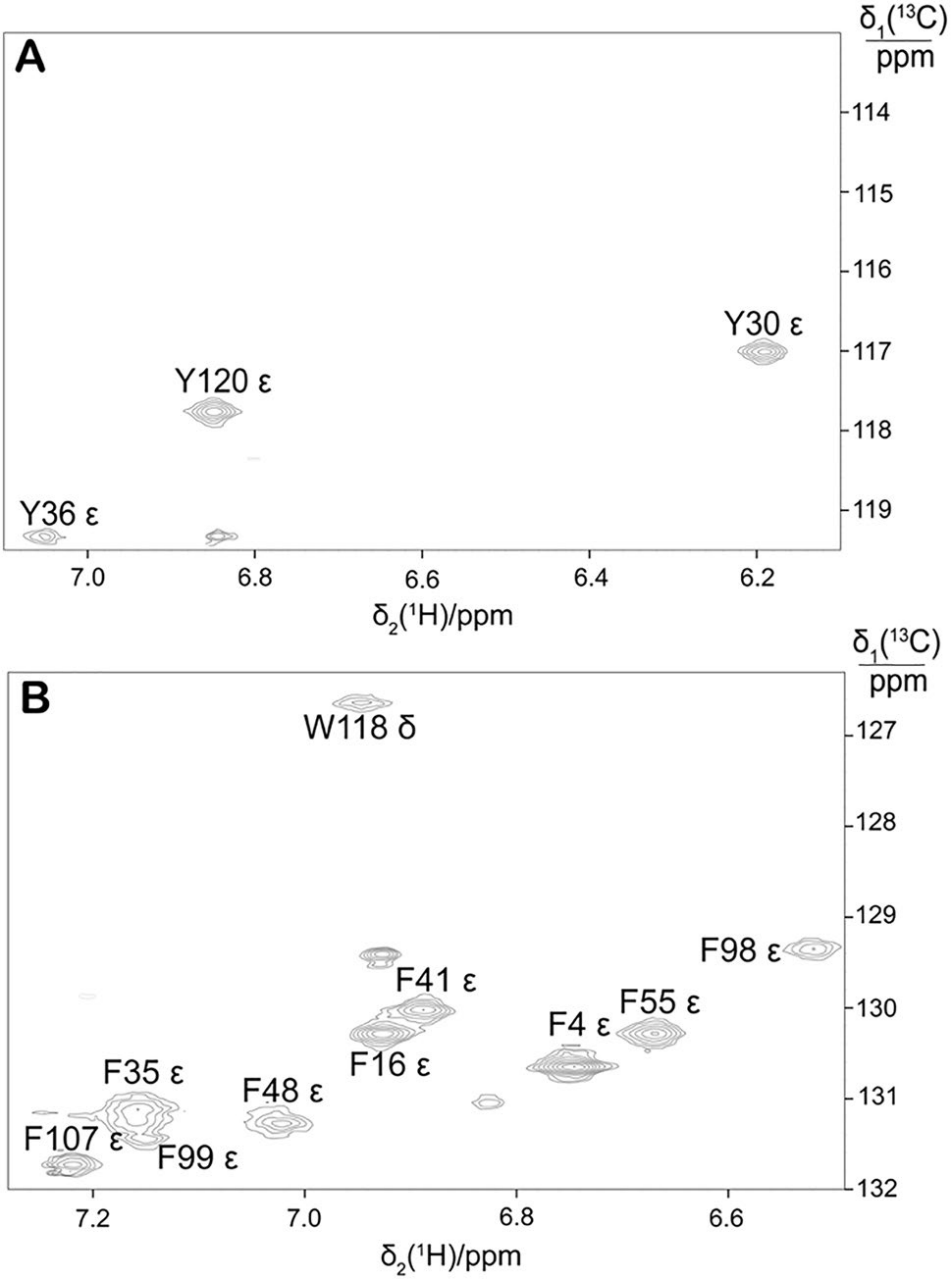
Selected spectral regions of the [^1^H,^13^C]-HSQC spectrum of PpiB produced by eCell CFPS with 2-^13^C glucose as the carbon source and with tyrosine, phenylalanine and tryptophan omitted and all other amino acids at 3.5 mM. The protein yield was 4.8 mg and the isotope labelling level about 45%. **A** C^ε^H cross-peaks of tyrosine and C^δ^H cross-peak of tryptophan. The weak unmarked cross-peak may arise from the C^δ^H group of His8, as the production of histidine from 2-^13^C glucose directs ^13^C to the δ position of the imidazole ring (Weininger 2017). **B** C^ε^H cross-peaks of phenylalanine. The two weak unmarked cross-peaks may arise from the C^ε^H groups of Phe27 and Phe123, for which no assignments have been reported

The biosynthetic pathways indicate that the ε carbons of Phe and Tyr and the δ_2_-carbon of Trp can be labelled also by using a different isotopologue precursor, 2-^13^C glucose. Using 1.5 g eCells in 25 mL CFPS buffer containing 30 mM 2-^13^C glucose yielded 4.8 mg PpiB with > 45% labelling degree of the ε carbons of Phe and Tyr. Figure 3 shows that this approach likewise avoided splittings by ^1^*J*_CC_ couplings.

**Fig. 4:**
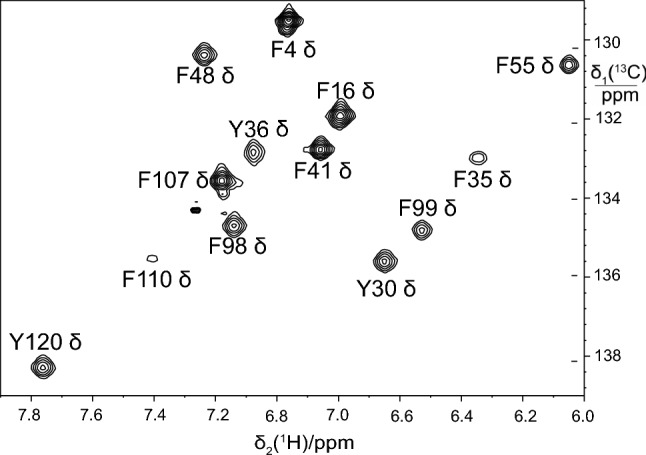
Selected spectral region of the [^1^H,^13^C]-HSQC spectrum of PpiB produced by eCell CFPS with 3-^13^C pyruvate and 4-^13^C erythrose as the carbon sources for Phe and Tyr. The yield was 1.6 mg purified protein, and the level of isotope labelling was > 70%

While 3-^13^C pyruvate enriches only one of two C^δ^ positions in the aromatic rings of Phe and Tyr, the biosynthetic pathways (Fig. 1) suggest that the additional provision of 4-^13^C erythrose will label both C^δ^ positions and thus increase the sensitivity of the NMR measurements. Using 1.5 g eCells in 25 mL buffer containing 33 mM 3-^13^C pyruvate and 30 mM 4-^13^C erythrose, 1.6 mg PpiB were obtained with a labelling level of 70%. The spectrum showed singlets in the ^13^C dimension as expected (Fig. 4).

## Discussion

Due to their relatively rigid and hydrophobic character, aromatic residues tend to be overrepresented in ligand binding sites and hydrophobic cores of proteins (Makwana and Mahalakshmi 2015). In enzymes, aromatic amino acids frequently form important interactions with substrates (Lanzarotti et al. 2011). This makes them attractive probes to assess 3D structure and ligand binding in pharmaceutical development and drug design (Brylinski 2018). To render aromatic side chains more amenable to monitoring by NMR spectroscopy, the spectral simplification afforded by atom-specific labelling confers an important benefit.

In contrast to protein production in vivo, where isotopic scrambling is best suppressed by using late metabolic precursors in isotope-labelled form together with auxotrophic bacterial strains, eCells provide a straightforward alternative route to atom-specific isotope labelling of aromatic amino acids starting from inexpensive and more readily available precursors. For example, the cost of isotope-labelled starting material for 4.8 mg PpiB produced from eCells was about 32 USD for 50 mg 2-^13^C glucose (Table 1). The equivalent sample produced in vivo would require much more isotope-labelled compound to satisfy the larger culture volumes involved. Typical amounts of ^13^C-labelled precursors used for expression in 1 L M9 minimal medium are 2.5 g ^13^C glucose, 2.0 g pyruvate or 1.3 g erythrose (Cai et al. 1998, 2019; Kasinath et al. 2013). The economics of eCell CFPS thus appear competitive to in vivo protein expression in minimal media also when measured in terms of cost of ^13^C-labelled precursor required per mg of isotope-labelled protein. We anticipate that actual outcomes will depend mostly on the intrinsic yields of the specific protein in eCell CFPS versus in vivo cell cultures.

Like in unlabelling experiments conducted in vivo (Rowlinson et al. 2022), provision of unlabelled amino acids in eCell CFPS allows suppressing undesired cross-peaks of the unlabelled amino-acid residues. This approach works well for discriminating between Phe and Tyr, as the *E. coli* metabolism does not easily swap carbon atoms between these two residue types (Takeuchi et al. 2007). The presence of tyrosine has been shown to differentially inhibit prephenate dehydrogenase (PDH; Hudson et al. 1983), thereby preventing 4-hydroxyphenyl pyruvic acid formation on the pathway to tyrosine (Fig. 1) and thus increasing the availability of the ^13^C-labelled precursors for the biosynthesis of other aromatic amino acids. Conversely, as PDH performs an irreversible catalytic step (Turnbull et al. 1990), unlabelled tyrosine is readily incorporated into the protein without diluting the pool of ^13^C-labelled intermediates. In a very similar way, prephenate dehydratase (PDT) is inhibited by as little as 50 µM phenylalanine (Gething et al. 1976). Finally, we found no evidence for eCell CFPS misdirecting ^13^C into non-targeted positions on the aromatic ring of the labelled amino acid. This establishes eCell CFPS as an attractive in vitro tool for selective labelling starting from inexpensive and readily accessible precursors.

**Table 1:** Comparison of precursors and their contribution to the cost of eCell expression for PpiB expression using 1.5 g eCells in 25 mL CFPS buffer^1^

Precursor/Energy source	Precursor cost (USD)	Labelling level (%) actual/max.	Protein yield (mg)	Cost per reaction	Position labelled in Phe and Tyr
3-^13^C pyruvate	$866/g	36/50	4.8	$36	δ
3-^13^C pyruvate + 1-^13^C glucose	$866/g + $282/g	50/100	2.7	$26	δ
3-^13^C pyruvate + 4-^13^C erythrose	$866/g + $1340/g	70/100	1.6	$104	δ
2-^13^C glucose	$305/g	46/50	4.8	$15	ε

^1^ Prices from Cambridge Isotope Laboratories (https://www.isotope.com) and Omicron Biochemicals, Inc. (https://www.omicronbio.com), Accessed 4 Jan 2023

The present work investigated the selective ^13^C enrichment of the δ or ε positions of aromatic amino acids by starting from different isotopologues. Like previous in vivo experiments conducted with selectively ^13^C-labelled glucose (Loquet et al. 2011), eCell CFPS achieves sparse ^13^C-labelling, where ^13^C enrichment can be targeted to the δ positions of Phe and Tyr residues using either the precursors 2-^13^C glucose (> 45%) or 3-^13^C pyruvate (> 36%). Even higher levels of ^13^C labelling in the δ position (> 70%) can be achieved by the combined use of 3-^13^C pyruvate and 4-^13^C erythrose. Previous efforts to label the δ positions of Phe and Tyr in vivo by the combined use of 1-^13^C glucose and ^13^C isotopologues of erythrose struggled to achieve labelling levels above 40% (Weininger 2017).

Some isotopic dilution due to a pool of unlabelled amino acids and precursors present in the eCells is difficult to avoid, as illustrated by our experiments conducted to label the δ positions of aromatic residues of PpiB by expression from 3-^13^C pyruvate and 1-^13^C glucose in eCells, where we obtained the expected labelling pattern (Figure S2) but a labelling level of only about 50%. Attempts to dialyse eCells in a large volume of buffer for an extended period to remove any unlabelled amino acids prior to eCell CFPS led to reduced protein yields, which may be expected as eCells gradually leak biomacromolecules (Van Raad and Huber 2021). Notably, however, our estimate of the labelling degree is conservative, as we compared the cross-peak volumes observed for the aromatic C–H groups with the cross-peak of ^13^C-pyruvate added to the solution as an internal standard. In a protein of the molecular weight of PpiB (19 kDa), transverse relaxation during the INEPT periods of the ^13^C-HSQC spectra would have affected the protein signals more than those of the internal standard.

In vivo experiments conducted with deuterated pyruvate and 4-^13^C erythrose have been shown to endow the δ positions of Tyr and Phe with single ^1^H-^13^C pairs with a labelling degree of 67% while simultaneously installing deuterium in neighbouring positions (Kasinath et al. 2013), which is particularly beneficial for ^13^C-relaxation measurements (Dreydoppel et al. 2021). While we did not perform experiments with deuterated compounds, we expect that the same labelling pattern would be obtained by eCell CFPS using the same precursors. This expectation is based on the result that eCells maintain the activities of the full complement of enzymes required for the biosynthesis of aromatic amino acids and thus perform in ways closely similar to live *E. coli*. For the same reason we anticipate that synthetic, commercially available late-stage precursors will perform as well in eCell CFPS as in in vivo protein expression.

## Conclusions

The eCell system allows the selective ^13^C-enrichment of aromatic groups from early precursors, including atom-selective labelling of ring positions without ^1^*J*(^13^C,^13^C) couplings. High levels of isotope incorporation can be achieved with little expense for isotope-labelled precursors. Facile control over the chemical environment in eCell CFPS enables the ‘unlabelling’ of specific residues by simple addition of the respective unlabelled amino acids. The preparation of eCells is straightforward, can easily be scaled up and makes costly dialysis membranes redundant. By preserving the activities of the enzymes required for multi-step anabolic amino-acid biosynthesis from inexpensive precursors, the eCell CFPS platform is highly attractive for selective isotope labelling of proteins for NMR spectroscopy.

## Supplementary Information

Below is the link to the electronic supplementary material.

Supplementary material 1 (PDF 792.7 kb)
